# Highly-ordered Triptycene Modifier Layer Based on Blade Coating for Ultraflexible Organic Transistors

**DOI:** 10.1038/s41598-019-45559-4

**Published:** 2019-06-24

**Authors:** Masaya Kondo, Takashi Kajitani, Takafumi Uemura, Yuki Noda, Fumitaka Ishiwari, Yoshiaki Shoji, Teppei Araki, Shusuke Yoshimoto, Takanori Fukushima, Tsuyoshi Sekitani

**Affiliations:** 1 The Institute of Scientific and Industrial Research, Osaka University, 8-1, Mihogaoka, Ibaraki, Osaka 567-0047 Japan; 2 Graduate School of Engineering, Osaka University, 2-1 Yamadaoka, Suita, Osaka 565-0871 Japan; 3 AIST-Osaka University Advanced Photonics and Biosensing Open Innovation Laboratory, AIST, 2-1 Yamada-Oka, Suita, Photonics Center P3 Bldg. 2-1, Osaka University, Osaka, 565-0871 Japan; 4 Laboratory for Chemistry and Life Science, Institute of Innovative Research, Tokyo Institute of Technology, 4259 Nagatsuta, Midori-ku, Yokohama 226-8503 Japan

**Keywords:** Molecular self-assembly, Electronic devices

## Abstract

We present a highly ordered surface modification layer for polymers based on ambient solution-processed triptycene (Trip) derivatives for high-mobility organic thin-film transistors (OTFTs). The nested packing of Trip molecules results in the formation of 2D hexagonal arrays, which stack one-dimensionally on the surface of polymer dielectrics without anchoring groups. The Trip surface was previously shown to be preferable for the growth of organic semiconductors (OSCs), and hence for enhancing the mobility of OTFTs. However, although the Trip modifier layer has been realized by thermal evaporation in a high-vacuum environment (TVE), it still has grain-boundary disorders that hinder the optimal growth of OSCs. To fabricate OTFTs with higher mobility, a disorder-free Trip layer is needed. We developed highly ordered Trip layers on polymer dielectrics via blade coating. In addition, we clarified that the highly ordered Trip modifier layer enhances the mobility of the OTFTs by more than 40%, relative to the disordered Trip layer prepared by TVE. Finally, we realized a ring oscillator composed of OTFTs with a highly ordered Trip layer.

## Introduction

Flexible skin-like device technologies have been developed for next-generation applications such as flexible bio-medical sensors and flexible physical sensors^1–3^. To build such devices, flexible transistors are used as active electronic elements^4–7^. Among the various options for realizing flexible transistors, organic thin-film transistors (OTFTs) are potential candidates because they are intrinsically flexible and are compatible with low-cost printing processes that can be applied to large-area plastic substrates^8–10^. In practice, fundamental logic and analog circuits can be built based on OTFTs^11^. Among the different types of OTFTs, those with polymer gate dielectrics are particularly promising because of their excellent flexibility and processability^12–14^.

However, the surfaces of polymer dielectrics have numerous disorders and trap sites because of the random orientation of the polymer chains. These disorders and trap sites are difficult to remove via conventional modification techniques such as the formation of self-assembled monolayers (SAMs)^15,16^, because of the lack of elements for anchoring the SAMs to the polymer surface. Therefore, it is challenging to form highly crystalline organic semiconductor (OSC) films on polymer dielectrics; consequently, OTFTs with polymer dielectrics usually exhibit a low field-effect mobility (hereafter referred to as “mobility”)^17,18^.

**Figure 1:**
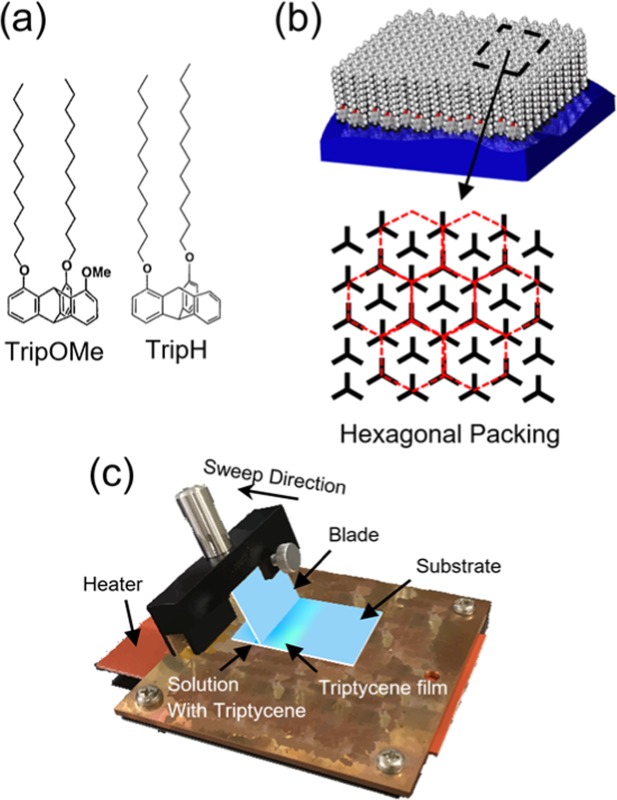
(**a**) Molecular structures of triptycene derivatives used in this work. (**b**) Triptycene film featuring 2D nested hexagonal packing. (**c**) Blade-coating unit used to fabricate triptycene films.

Recently, it has been reported that specific triptycene (*e*.*g*., TripOMe, Fig. 1a, left) layers are useful for modifying polymer surfaces to mitigate the negative effect arising from disorders and trap sites^19^. The Trip molecules arrange themselves regularly to form a 2D nested hexagonal packing and 1D layer stacking on polymer surfaces without any anchoring groups, as shown in Fig. 1b ^20^. A Trip film was formed by thermal vacuum evaporation (TVE) processes on polymer gate dielectrics, and OTFTs with the modified gate dielectrics showed improved mobility^19^. However, the general mechanism of thin-film formation by TVE depends on simultaneous nucleus formation and growth. Hence, the thin-films still possess grain boundaries that contribute to the defects that prevent the growth of organic semiconductors (hereafter referred to as OSCs).

In addition, in terms of process cost and applicability to large-area devices, the formation of a uniform Trip layer in an ambient environment is important. However, the formation of highly uniform Trip films has proved challenging when using general ambient solution processes such as drop casting and spin coating, as shown in the supporting information corresponding to Supplementary Fig. S1. The adhesion of a Trip layer to the substrate depends on the physical adsorption. Therefore, in the case of spin coating, there are insufficient Trip molecules remaining on the substrate and hence, a uniform layer cannot be formed. In drop casting, film formation depends on the random self-organization of Trip, leading to non-uniformity. As a result, drop-cast Trip films exhibited increased dispersion of the enhanced mobility while spin-coated Trip films produced no enhancement in the mobility.

Herein, we report on a highly ordered Trip layer and the enhancement of its effect as a modifier layer, based on a solution process. The highly ordered Trip layer is realized by blade coating (BC), which is a meniscus-guided solution process^21,22^. The BC unit is shown in Fig. 1c. BC is a simple solution process performed under ambient conditions. In this study, we demonstrated that Trip (TripOMe and TripH, Fig. 1a) films formed by BC are highly ordered and enhance the mobility of OTFTs, relative to those fabricated by TVE. We fabricated OTFTs with hybrid gate dielectrics of ultrathin parylene (38-nm diX-SR) and a Trip layer (6 nm). Parylene is a polymer, which is well suited for the gate dielectrics of OTFTs. Because parylene is formed by chemical vapor deposition (CVD), it is formed in a low-temperature and inert process^23^. Moreover, OTFTs with parylene gate dielectrics exhibit excellent flexibility^24^. The preparation of the Trip layer using BC produced a two-dimensional, smooth, and highly-ordered Trip surface on parylene, leading to a higher mobility relative to that based on TVE. We mainly used TripOMe in the present study, while TripH served as a reference to show the applicability of BC to other types of Trip. Further, to demonstrate the advantage of the higher mobility, ultraflexible ring oscillators with TripOMe were fabricated by both the TVE and BC methods and their performances were compared. All the experimental conditions are described in detail in the Method section.

**Figure 2:**
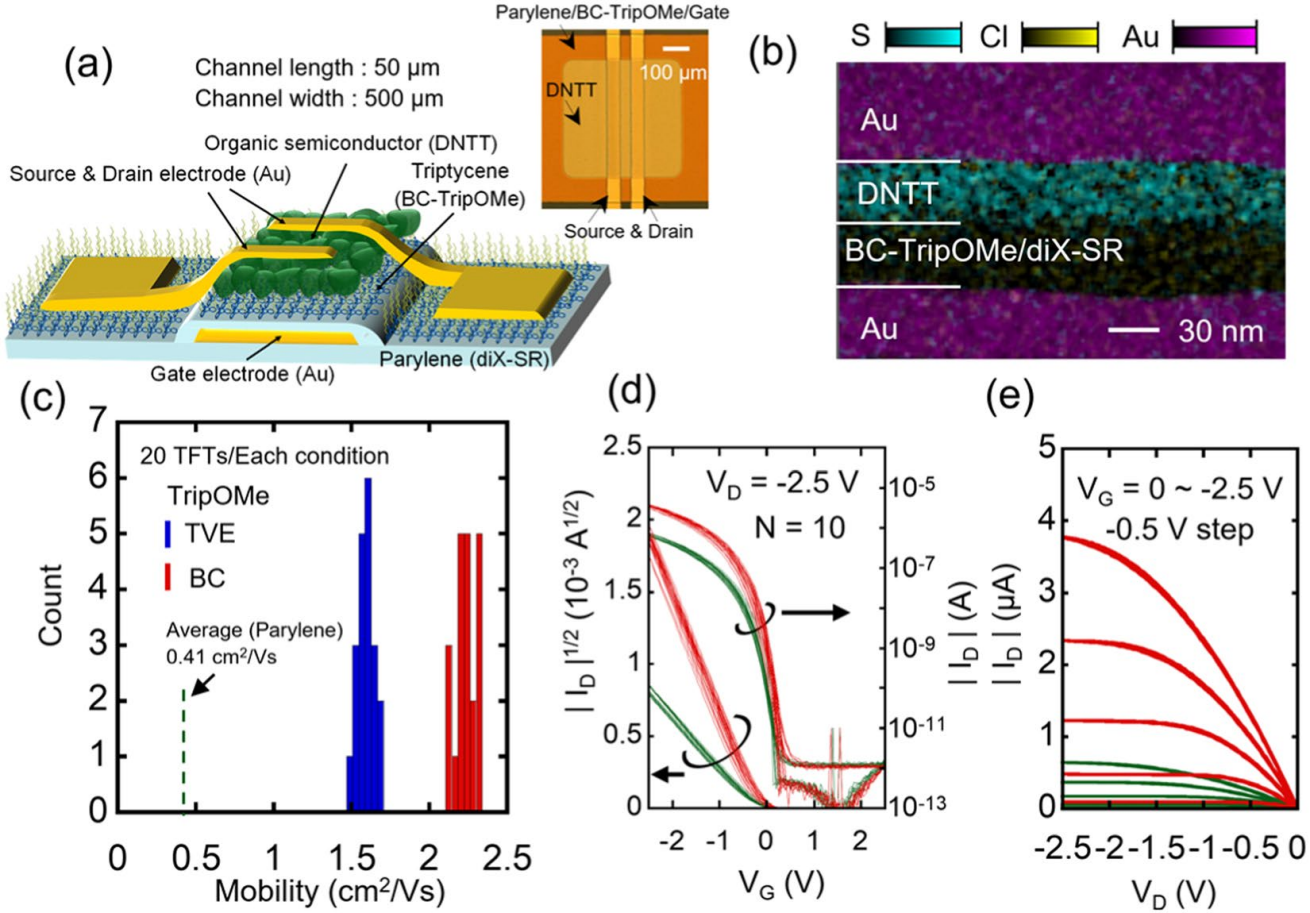
Structures and characteristics of organic thin-film transistors (OTFTs) modified by TripOMe. (**a**) Schematic and optical micrograph of an OTFT. (**b**) Cross-sectional energy-dispersive X-ray spectroscopy image captured by a transmission electron microscope. (**c**) Histograms of the field-effect mobility of the OTFTs. The blue histograms show OTFTs with TripOMe films formed by thermal vacuum evaporation (TVE-TripOMe). The red histograms show OTFTs with TripOMe films formed by blade coating (BC-TripOMe). (**d**,**e**) Transfer and output characteristics of OTFTs with BC-TripOMe (red curves) and of OTFTs with pristine parylene (green curves).

Figure 2a,b shows a cross-sectional diagram, optical micrograph, and transmission electron microscopy-energy dispersive X-ray spectroscopy (TEM-EDX) image of an OTFT. All OTFTs possess the top-contact/bottom-gate structure and have dinaphto[2,3-b:29,39-f]thieno[3,2-b]thiophene (DNTT) as their active layer (Fig. 2a). The TEM-EDX image of an OTFT shows clear boundaries between the material layers. Approximately 6-nm Trip layers were formed on the parylene dielectrics by TVE and BC to compare their OTFT performances. Hereafter, these two Trip films are referred to as TVE- and BC-Trip. The thicknesses of the parylene and Trip derivatives were estimated from the capacitance of the metal/dielectric/metal structures. In the metal/parylene/metal, metal/TVE-Trip/parylene/metal, and metal/BC-Trip/parylene/metal structures, the averages of the capacitance were 72.3, 62.2, and 62.9 nF/cm^2^, respectively. Based on these values, we estimated the thicknesses of the parylene, TVE-Trip, and BC-Trip to be 38.0, 6.2, and 5.8 nm, respectively.

We compared the mobility of OTFTs fabricated with TVE-Trip and with BC-Trip. The blue histogram in Fig. 2c shows the mobility of OTFTs with the TVE-TripOMe layer. The transfer and output characteristics are shown in Supplementary Fig. S2, and indicate negligible hysteresis. The average mobility of the OTFTs with pristine parylene (as a reference device) is 0.41 cm^2^ V^−1^ s^−1^, whereas, that of OTFTs with TVE-TripOMe is 1.59 ± 0.05 cm^2^ V^−1^ s^−1^. The red histogram in Fig. 2c shows the mobility of OTFTs with the BC-TripOMe layer. The transfer and output characteristics are shown in Fig. 2d,e. The average mobility is 2.23 ± 0.07 cm^2^ V^−1^ s^−1^. Comparative experiments were performed using the same process batches and the same material batches, except for the formation process of Trip film. OTFTs with BC-TripOMe exhibited a 40% higher mobility than those fabricated with TVE-TripOMe. Moreover, the variation in the threshold voltages of the OTFTs with TVE- and BC-TripOMe was similar. Recently, it has been reported that erroneous mobility extraction for OTFTs causes its overestimation or underestimation. To prevent such misleading extraction, the reliability factor (r) was proposed by Choi *et al*. to assess the validity of mobility extraction, where r can exceed 75% in high-quality OTFTs^25^. In addition, Xu *et al*. calculated r for OTFTs with various structures and checked their validity^26^. In the present study, we calculated r to check the validity of our extraction; the average values for the TVE-TripOMe and BC-TripOMe samples were 89 ± 1 and 88 ± 1%, respectively, based on the calculation shown in supporting information corresponding to Supplementary Fig. S3. These values suggest that the mobility extraction is reliable.

**Figure 3:**
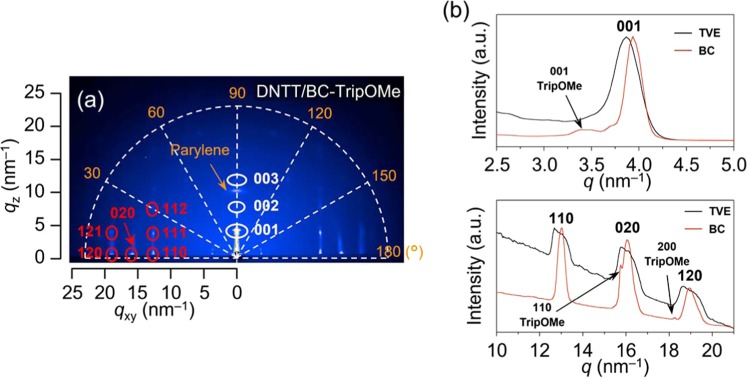
Grazing incidence X-ray diffraction (GI-XRD) of DNTT on TripOMe. (**a**) 2D GI-XRD image of 30-nm-thick DNTT film on BC-TripOMe film. (**b**) 1D GI-XRD profiles of 30-nm-thick DNTT film on TVE-TripOMe film (black line) and BC-TripOMe (red line).

We examined the reason for the BC-TripOMe layer producing a higher mobility than the TVE-TripOMe layer. First, we examined the differences in the DNTT films on parylene with/without TripOMe. Figure 3a shows two-dimensional grazing-incidence X-ray diffraction (2D GI-XRD) images of DNTT/BC-TripOMe/parylene. The 2D GI-XRD results for the DNTT film on pristine parylene are shown in Supplementary Fig. S4. The diffraction spots were indexed as the (001), (110), (111), (020), and (120) planes of the DNTT assembly, based on a unit cell belonging to the monoclinic *P2*_1_ space group. Supplementary Table S1 lists the detailed cell parameters. From the azimuthal-angle dependency, the full-width at half maximum (FWHM) of the diffraction from the (001) plane was found to be smaller than that observed for DNTT/parylene, as shown in Supplementary Fig. S5. The small FWHM indicates that DNTT has superior crystallinity, that is, fewer disorders, on BC-TripOMe/parylene than on parylene alone. In addition, the formation of large crystalline structures in semiconducting DNTT films was confirmed by atomic force microscopy (AFM). The AFM images in Supplementary Fig. S6a,b clearly show that the overall grain size of DNTT on BC-TripOMe is larger than that on pristine parylene. Thus, the larger and disorder-less DNTT grain on TripOMe could be the main reason for those OTFTs with TripOMe exhibiting superior mobility to those with pristine parylene.

Second, we examined the differences between DNTT films on BC- and TVE-Trip. In the first step, we compared AFM images of DNTT on BC- and TVE-TripOMe, as shown in Supplementary Fig. S6b,c. However, the mobility difference could not be explained based on the difference in the grain size of the DNTT, because the grain sizes were found to be identical. Thus, in the second step, we conducted GI-XRD on a DNTT film on BC-TripOMe/parylene and TVE-TripOMe/parylene. The 2D GI-XRD results for the DNTT film on TVE-TripOMe/parylene are shown in Supplementary Fig. S7. However, based on the azimuthal-angle dependency, we could not find any difference in the FWHM of the diffraction from the (001) plane. Therefore, in the third step, we performed a detailed analysis of the 1D out-of-plane and in-plane diffraction peak profiles of DNTT on both BC- and TVE-TripOMe (Fig. 3b). The profiles revealed that the DNTT/BC-TripOMe film exhibits much sharper diffraction peaks from out-of-plane and in-plane than the DNTT/TVE-TripOMe film. The results of this detailed analysis indicates that the crystal integrity of DNTT on BC-TripOMe was more enhanced than that on TVE-TripOMe. In other words, DNTT on BC-TripOMe could have fewer disorders than the TVE-TripOMe, leading to a higher degree of mobility. Moreover, we found that the diffraction peak was slightly shifted in Fig. 3b. This shift reflects the differences in the DNTT crystal lattices on BC- and TVE-TripOMe, as shown in Supplementary Table S1. The shift in the crystal lattice might affect the packing of DNTT, leading to there being fewer disorders.

**Figure 4:**
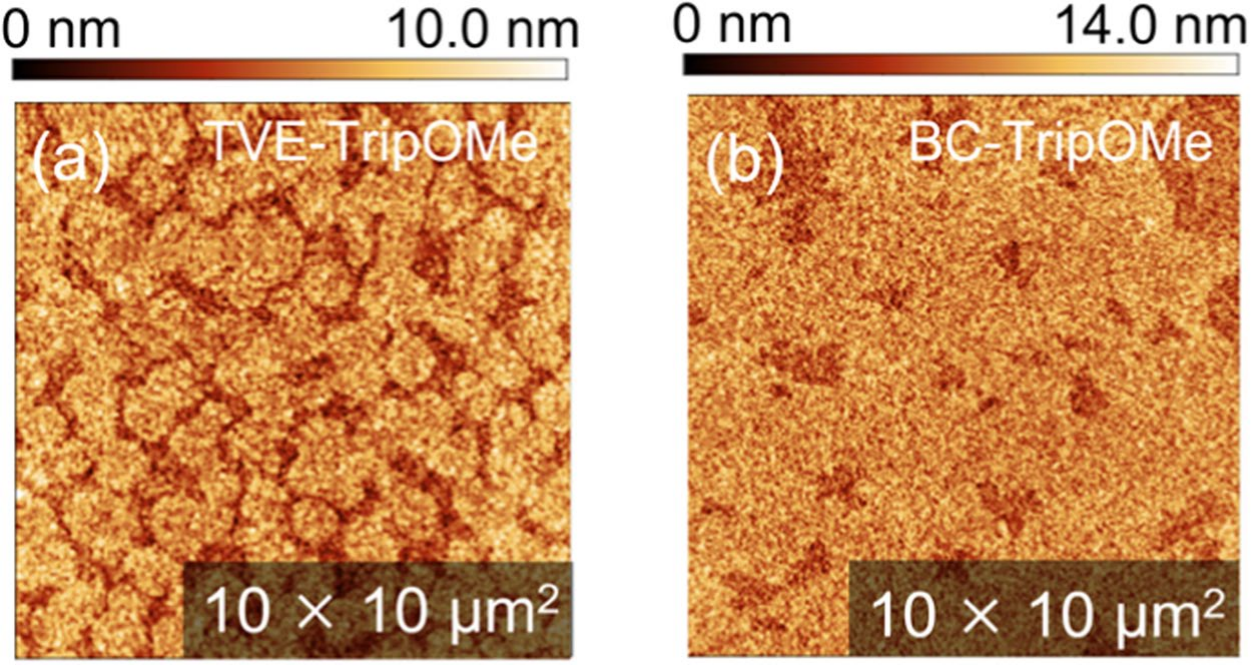
Morphologies of TripOMe films. (**a**) TVE-TripOMe. (**b**) BC-TripOMe.

Finally, we discussed the origin of the difference in the DNTT between TVE- and BC-TripOMe. The origin of one of the preferable DNTT crystallites on BC-TripOMe can be explained by the morphology of the TripOMe films. The growth of OSCs is strongly influenced by the surface morphology and surface energy of the substrate^27–29^. We observed the morphology of the TripOMe films as shown in Fig. 4. Molecular terraces unambiguously extend broadly in BC-TripOMe. Compared with the TVE-Trip surface, BC-trip has fewer grain boundaries as well as a highly ordered surface, thus could avoid deterioration of the crystallinity of OSCs. In addition, BC-TripOMe has the same surface energy as TVE-TripOMe (Supplementary Table S2). Therefore, the results of the present study could indicate that the uniform surface of the BC-Trip could be the origin of the mobility enhancement.

Another reason could be the preferable packing of the Trip layer. Meniscus-guided OSCs possess a high mobility due to the change in the crystal packing^30–32^. Thus, in this analogy, the BC-TripOMe also could possess more packing that is preferably closed to the growth of DNTT than TVE-TripOMe. Previous studies have found that the phase states, that is, the packing of SAMs, influence the growth of OSCs^33,34^. However, because both TripOMe films were too thin, we could not acquire diffraction peaks that were sufficiently strong to determine the crystal lattices, as shown in Supplementary Fig. S8. Therefore, we could not evaluate the packing effect of Trip.

The highly ordered Trip layer prepared via BC is attributed to the fixed starting points and the direction of the crystal nuclei, formed from the blade-guided solution. BC utilizes the surface tension of a solution held between the blade surface and substrate surface. Such surface tension fixes the contact line of the solution and forces molecules to be aligned in a preferable order^30–32^. The BC process can be applied to another type of Trip, namely, TripH (Fig. 1a, right). Although TripH lacks only a methoxy group on the main skeleton, relative to TripOMe, TripH is difficult to arrange on a 2D films. Therefore, TripH formed a rough and disordered surface with a large height step of around 10 nm for TVE. On the other hand, the use of the BC process dramatically improved the surface morphology of the TripH. As well as TripOMe, OTFTs with BC-TripH exhibited higher mobility than those with TVE-TripH. Interestingly, we noted that TripH did not improve the mobility of OTFTs, even if BC-TripH possessed a surface morphology and surface energy that were equivalent to those of BC-TripOMe. These results imply that TripH could present packing that is disadvantageous to the growth of OSCs, although the molecular structure is almost the same as that of TripOMe. The detailed results are discussed in the Supporting Information for Supplementary Figs S9–12.

High mobility is crucial for realizing fast operating logic and analog circuits. We demonstrated that OTFTs modified with BC-TripOMe have a higher mobility than those modified by TVE-TripOMe and applied the process to a relatively large device. We manufactured ultraflexible organic ring oscillators on 4 × 5 cm^2^ flexible films. Although the substrates and utilized OTFTs are larger than those shown in Fig. 2, BC-TripOMe can nevertheless be applied. Supplementary Fig. S13 shows the characteristics of OTFTs arranged on the same substrates as the oscillators. The channel length and width are 10 µm and 16 mm, respectively. The extracted mobility is consistent with that shown in Fig. 2c. This result indicates that BC-TripOMe possesses scalability.

**Figure 5:**
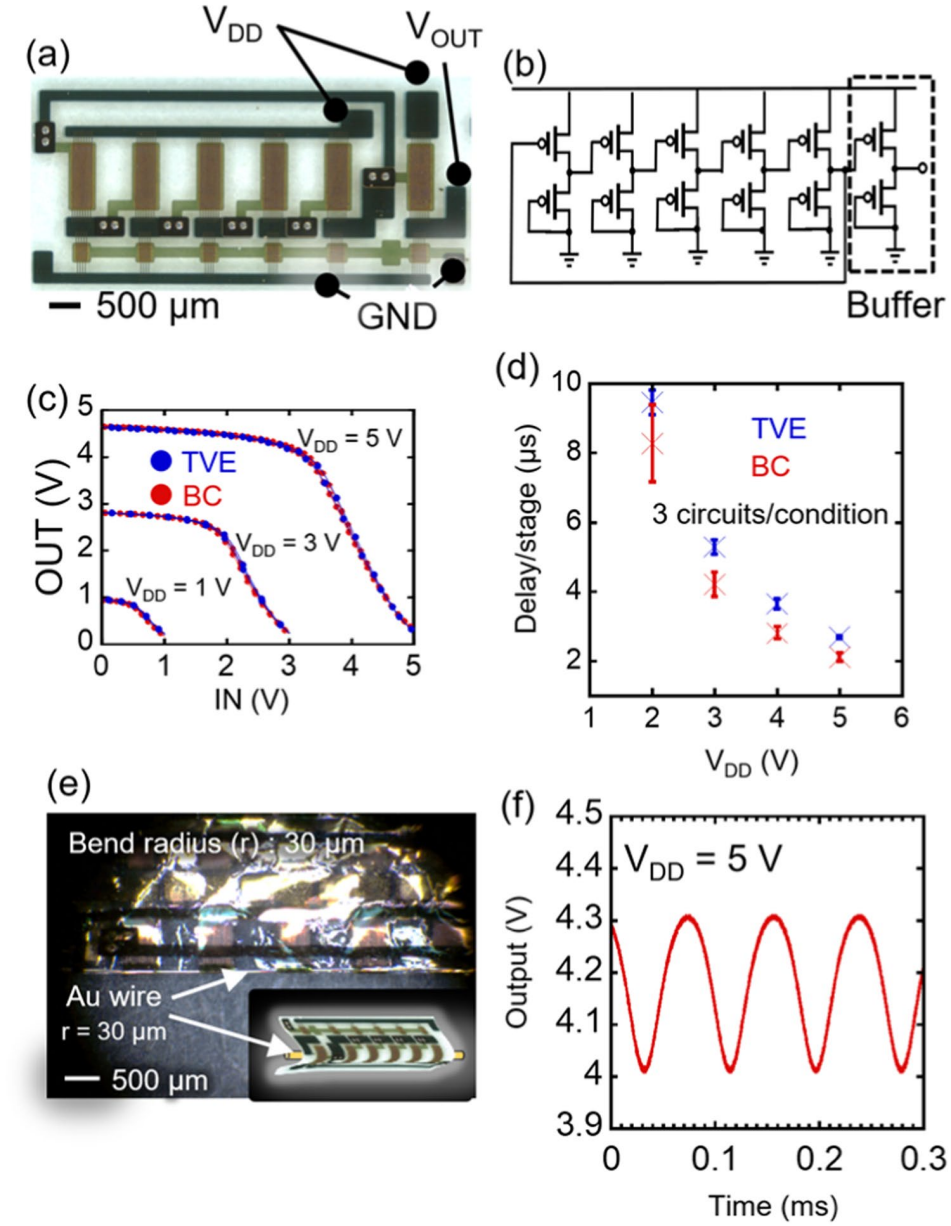
Organic ring oscillator with a TripOMe modification layer. (**a**) Optical micrograph of a ring oscillator. (**b**) Circuit diagram. (**c**) Transfer curves of diode-load inverters. Blue curves represent the performance of an inverter with TVE-TripOMe. Red curves depict the performance of an inverter with BC-TripOMe. (**d**) Signal delay/stage of ring oscillators with a TVE-TripOMe (Blue cross) and BC-TripOMe (Red cross). (**e**) Photograph of a ring oscillator rolled onto a Au wire, 30 µm in radius. (**f**) Output wave of a ring oscillator after rolling up on a 30-µm-radius Au wire.

We compared the ring oscillator characteristics and demonstrated that the oscillators with BC-TripOMe exhibited a higher mobility while the variation was equivalent to that with TVE-TripOMe. The optical micrograph and circuit diagram of the ring oscillator circuit are shown in Fig. 5a,b, respectively. Each ring oscillator consists of five-stage diode-load inverters with a single buffer stage, which contains 12 OTFTs. Figure 5c shows the operation of single-stage diode-load inverters with BC and TVE-TripOMe. Supplementary Fig. S14 shows the typical output signal of a ring oscillator being operated at a low supply voltage (*V*_DD_) of 5 V and one example with BC-TripOMe oscillating at 41.4 kHz, indicating that the signal delay per stage is 2.12 µs. The ring oscillators consist of diode-load inverters, such that the output voltage amplitude is smaller than *V*_DD_. To the best of our knowledge, the operating speed at a supply voltage of 5 V is the fastest among organic ring oscillators, based on polymer-gate dielectrics. Figure 5d compares the signal delays per stage of the ring oscillators for TVE- and BC-TripOMe. Clearly, the delay per stage of the BC-type is smaller than that of the TVE-type. This result points to OTFTs with BC-TripOMe having a higher mobility. Moreover, although the delays were averaged for each of the three devices on 4 × 5 cm^2^ flexible films, the dispersion of the ring oscillators with BC-TripOMe is equivalent to that with TVE-TripOMe. In the other words, a total of 36 OTFTs with BC-TripOMe in the three oscillators would have a dispersion that was equivalent to those with TVE-TripOMe. This result also points to the BC-TripOMe film maintaining a high level of uniformity and scalability. Further, we demonstrated that the TripOMe/parylene hybrid gate dielectrics have high flexibility by bending a ring oscillator (Fig. 5e,f). The device was encapsulated in 1-µm parylene, set in a neutral-strain position. It was then rolled onto a Au wire, 30 µm in radius, as shown in Fig. 5e and the inset. Figure 5f shows the operation of the ring oscillator after bending. When bent by 30 µm, the device oscillates at 11 kHz, indicating that the Trip/parylene hybrid gate dielectrics have high flexibility.

In conclusion, we developed highly ordered Trip films on ultraflexible polymer dielectrics based on BC, to investigate the practicality of manufacturing OTFTs with high mobility while operating at a low voltage. We demonstrated that the highly ordered Trip modification layer enhanced the mobility of the OTFTs by over 40%, relative to OTFTs with a disordered Trip layer. We found that the origin of this improvement is the crystallinity enhancement of the OSCs on the highly ordered BC-Trip layers. Measurements of ultraflexible organic ring oscillators with TripOMe revealed faster signals per stage in organic ring oscillators with the highly ordered TripOMe than those with TVE-TripOMe. The oscillation frequency was the highest among the organic ring oscillators with polymer-gate dielectrics. Finally, the developed highly ordered Trip modification layer was applied to ultraflexible OTFTs and circuits. A disorder-free surface is also a prerequisite in other research fields; hence, we expect that the highly ordered Trip layer will be widely applicable.

## Methods

### Materials

Unless otherwise noted, all commercial reagents were used as received. TripOMe^20^ and 1,8-dihydroxytriptycene^35^ were prepared according to previously reported procedures and were unambiguously characterized by NMR spectroscopy and atmospheric pressure chemical ionization time-of-flight (APCI-TOF) mass spectrometry. Dinaphto[2,3-b:29,39-f]thieno[3,2-b]thiophene (DNTT) was purchased from Nippon Chemical Industrial Co., Ltd. Parylene was provided by Daisan Kasei as diX-SR.

### Synthesis of TripH

Under argon, 1-bromododecane (2.7 mL, 11.2 mmol) was added at 25 °C to a *N*,*N*-dimethylformamide (DMF) solution (100 mL) of a mixture of 1,8- dihydroxytriptycene (1.0 g, 3.72 mmol) and K_2_CO_3_ (2.04 g, 14.9 mmol), and the resulting mixture was stirred at 75 °C for 24 h. After being allowed to cool to 25 °C, the reaction mixture was poured into water and extracted with CH_2_Cl_2_. A combined organic extract was washed with brine, dried over anhydrous MgSO_4_, and then evaporated to dryness under reduced pressure. The residue was subjected to column chromatography on SiO_2_ (CHCl_3_/hexane 1/2 v/v) to allow isolation of TripH as a white solid (2.08 g, 3.35 mmol) in 90% yield: ^1^H NMR (500 MHz, CDCl_3_): *δ* (ppm) 7.41–7.39 (m, 1 H), 7.36–7.34 (m, 1 H), 7.00 (d, 1 H, *J* = 7.2 Hz), 6.96–6.95 (m, 2 H), 6.89 (dd, *J* = 8.2, 7.2 Hz, 2 H), 6.56 (d, *J* = 8.2 Hz, 2 H), 6.39 (s, 1 H), 5.38 (s, 1 H), 4.01–3.92 (m, 4 H), 1.88–1.82 (m, 4 H), 1.60–1.51 (m, 4 H), 1.44–1.24 (m, 34 H), 0.88 (t, *J* = 6.78 Hz, 6 H). ^13^C NMR (125 MHz, CDCl_3_): *δ* (ppm) 154.17, 148.13, 146.50, 145.66, 133.72, 125.82, 125.01, 124.85, 123.91, 123.60, 116.50, 110.09, 68.88, 54.59, 40.24, 32.10, 2.992, 29.91, 29.86, 29.72, 29.69, 29.55, 26.31, 22.86, 14.28. FT-IR (KBr): *ν* (cm^–1^) 2923, 2853, 1599, 1486, 1397, 1378, 1472, 1279, 1195, 1107, 1073, 789, 760, 740, 721. APCI-TOF mass: calcd. for C_44_H_63_O_2_ [M + H]: *m*/*z* = 623.4828; found: 623.4819.

### Preparation of triptycene films

Triptycene films were formed by TVE and BC. TVE was carried out using an ULVAC EX200, and the film thickness was monitored using a quartz-crystal microbalance. BC was carried out using a custom-made deposition system in ambient air. The triptycene powder was dissolved in mesitylene (Wako chemicals) to obtain a 0.5 mM solution. The blade was made of glass. All substrates were heated to 50–60 °C with a silicone-rubber heater while depositing the triptycene/mesitylene solution. The blade speed was fixed to between 40 and 50 µm/s. In the present study, the TVE-Trip process required approximately 1 h, including the evacuation and cooling time, while the BC-Trip process required approximately 8.5 or 17 min for substrate areas of 2 × 3 cm^2^ and 4 × 5 cm^2^, respectively. After forming triptycene films by TVE and BC processes, all the substrates were annealed at 120 °C for 1 h under vacuum at around 100 Pa.

### Surface energy measurements

First, 1.5-µm parylene films were formed as substrates. One substrate was used as a reference. Then 6-nm TripH and TripOMe were thermally evaporated onto 1.5-µm parylene films. For other substrates, TripH and TripOMe were blade-coated onto 1.5-µm parylene films. The blade coating process is described in *Preparation of triptycene films*. The surface energies of the films were evaluated based on contact angles. Ethylene glycol (Wako Pure Chemical Industries Ltd.) and Milli-Q water droplets were used as solvents. The droplet volume was fixed at 2 µl. The contact angles were measured using a FAMAS *FACE* Measurement & Analysis System. The contact angles were measured at three different positions on each film and the average surface energies were calculated based on the values thus obtained. The surface energies were calculated by the Owens-Wendt-Kaelble approach^36^. The polar and dispersive components were taken from a previous report^37^. (Ethylene glycol: 17.6 and 30.1 mJ m^−2^, respectively; Milli-Q water: 50.7 and 22.1 mJ m^−2^, respectively.)

### Fabrication and measurement of OTFTs

A 30-nm Au layer was thermally evaporated onto 1.5-µm parylene supported on a glass film through a shadow mask to serve as the gate electrode. The substrate areas were 2 × 3 cm^2^. Next, before the deposition of parylene as the gate dielectric, the Au surface was treated with oxygen plasma at 100 W for 3 min. Then, a 38-nm parylene layer was deposited by CVD. To modify the surface of the parylene film, approximately 6-nm Trip derivative films were prepared according to aforementioned methods. Finally, 30-nm DNTT and 50-nm Au layers were thermally deposited through a shadow mask to form the active layer and source and drain electrodes, respectively. The channel length and width of the OTFTs were 50 and 500 µm, respectively. All the electrical characterizations were conducted in ambient air in a dark room.

### Synchrotron radiation X-ray diffraction experiments

The grazing incidence X-ray diffraction (GI-XRD) images were obtained using the BL45XU beamline at Spring-8 (Hyogo, Japan) equipped with a Pilatus3X 2 M (Dectris) detector. The scattering vector, *q* = 4*π*sin*θ/λ*, and the position of the incident X-ray beam on the detector were calibrated using several orders of layer reflections from silver behenate (*d* = 58.380 Å), where, *2θ* and *λ* refer to the scattering angle and wavelength of the X-ray beam (1.0 Å), respectively. The sample-to-detector distance was 0.3 m. The obtained diffraction images were integrated along the Debye–Scherrer ring to afford 1D intensity data using the FIT2D software^38^. The lattice parameters were refined using CellCalc v.2.10^39^.

### Organic ring oscillator

We used a 1-μm parylene as substrate supported on a glass film. The channel was formed using a fine shadow mask. The channel length of the OTFTs in a ring oscillator was 10 µm. In Fig. 5c,d and S14, we used ring oscillators in which the ratios of the driver to load OTFTs were 6:1. We evaluated the devices without encapsulation and delamination from the supporting glass. In bending as shown in Fig. 5e,f, we used another ring oscillator for which the ratio of the driver to the load OTFTs was 8:1. Before the bending test, the oscillator was encapsulated in a 1-µm layer of parylene.

## Supplementary information

Supplementary Material
