# Supplementary material for: Highly-ordered Triptycene Modifier Layer Based on Blade Coating for Ultraflexible Organic Transistors
Source: Sci Rep. 2019 Jun 24;9:9200. doi: 10.1038/s41598-019-45559-4 (PMC6591239; doi:10.1038/s41598-019-45559-4)
Supplement: Supplementary file 1 — Supplementary Material [file 41598_2019_45559_MOESM1_ESM.pdf]

Supplementary Material

Title: Highly-ordered Triptycene Modifier Layer Based on Blade Coating for Ultraflexible Organic Transistors

Masaya Kondo, Takashi Kajitani, Takafumi Uemura, Yuki Noda, Fumitaka Ishiwari, Yoshiaki Shoji, Teppei Araki, Shusuke Yoshimoto, Takanori Fukushima*, Tsuyoshi Sekitani**

Masaya Kondo, Prof. Takafumi Uemura, Dr. Yuki Noda, Dr. Teppei Araki, Dr. Shusuke Yoshimoto, Prof. Tsuyoshi Sekitani
The Institute of Scientific and Industrial Research, Osaka University
8-1, Mihogaoka, Ibaraki, Osaka 567-0047, Japan
E-mail: sekitani@sanken.osaka-u.ac.jp

Masaya Kondo, Dr. Teppei Araki, Prof. Tsuyoshi Sekitani
Graduate School of Engineering, Osaka University, 2-1 Yamadaoka, Suita, Osaka 565-0871, Japan
E-mail: sekitani@sanken.osaka-u.ac.jp

Masaya Kondo, Prof. Takafumi Uemura, Dr. Teppei Araki, Prof. Tsuyoshi Sekitani
AIST-Osaka University Advanced Photonics and Biosensing Open Innovation Laboratory, AIST, 2-1 Yamada-Oka, Suita, Photonics Center P3 Bldg. 2-1, Osaka University, Osaka 565-0871, Japan
E-mail: uemura-t@sanken.osaka-u.ac.jp

Prof. Takashi Kajitani, Dr. Fumitaka Ishiwari, Prof. Yoshiaki Shoji, Prof. Takanori Fukushima
Laboratory for Chemistry and Life Science, Institute of Innovative Research, Tokyo Institute of Technology, 4259 Nagatsuta, Midori-ku, Yokohama 226-8503, Japan.
E-mail: fukushima@res.titech.ac.jp

Keywords: organic field-effect transistors, flexible devices, field-effect mobility, triptycene derivatives, highly-ordered nanolayer.

Comparison of spin-coated and drop-casted TripOMe

We produced TripOMe using both the drop-casting and spin-coating processes. To fabricate the OTFTs, a 30-nm Au layer was thermally evaporated onto 1.0 μm parylene film supported on a glass film through a shadow mask to serve as the gate electrode. The substrate areas were 2 × 3 cm^2. Next, before the deposition of parylene as the gate dielectric, the Au surface was treated with oxygen plasma at 100 W for 3 min. Then, approximately 30 nm of parylene was deposited by CVD. For the Trip formation based on the spin-coating and drop-casting processes, the TripOMe powder was dissolved in mesitylene (Wako chemicals) to give a 0.5 mM solution. For the spin coating, the first spin speed was 500 rpm which was maintained for 5 s. In the next step, the spin speed was increased to 3000 rpm and held for 20 s. In the drop-casting process, 100 µL of solution was cast to cover the entire parylene surface. After the Trip formation, all the substrates were annealed at 120 °C for 1 h under a vacuum at around 100 Pa. Finally, 30-nm DNTT and 50-nm Au layers were thermally deposited through a shadow mask to form the active layer and source and drain electrodes, respectively. The channel length and width of the OTFTs were 50 and 500 µm, respectively.

Supplementary Figure S1a-c shows the transfer characteristics of OTFTs with pristine parylene, spin-coated TripOMe and drop-casted TripOMe, respectively. The average mobility of the OTFTs with pristine parylene as a reference device were 0.46 ± 0.01 $cm^2\ V^{-1}\ s^{-1}$; whereas, OTFTs with spin-coated TripOMe did not exhibit any enhancement in their mobility, relative to those with pristine parylene, as shown in Supplementary Figure S1a,b. The average mobility was 0.25 cm^2/Vs in those OTFTs with the spin-coated TripOMe. We found that the spin-coated TripOMe did not exhibit increased capacitance relative to that with pristine parylene. This implies that spin coating cannot form a Trip film with enough thickness to improve OTFTs performance. In contrast, OTFTs with drop-cast TripOMe

exhibited an enhanced level of mobility of 1.92 ± 0.47 cm^2 V^{-1} s^{-1}. However, the characteristics deviated considerably, as shown in Supplementary Figure S1c. This is because the quality of the drop-cast film depends on the random drying of the solution. This random drying prevents the formation of a uniform Trip film.

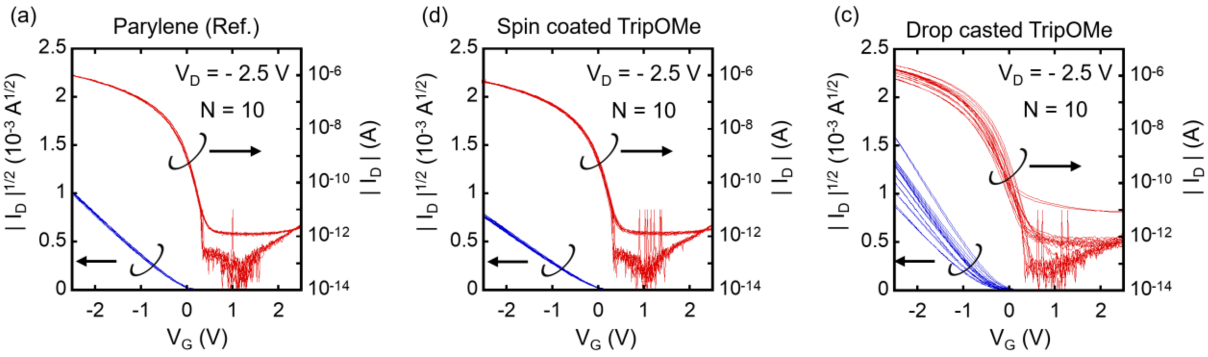

Figure S1. Comparison of OTFT formation using spin-coated and drop-casted TripOMe. Transfer characteristics of OTFTs with a) pristine parylene, b) spin-coated TripOMe, and c) drop-cast TripOMe, respectively.

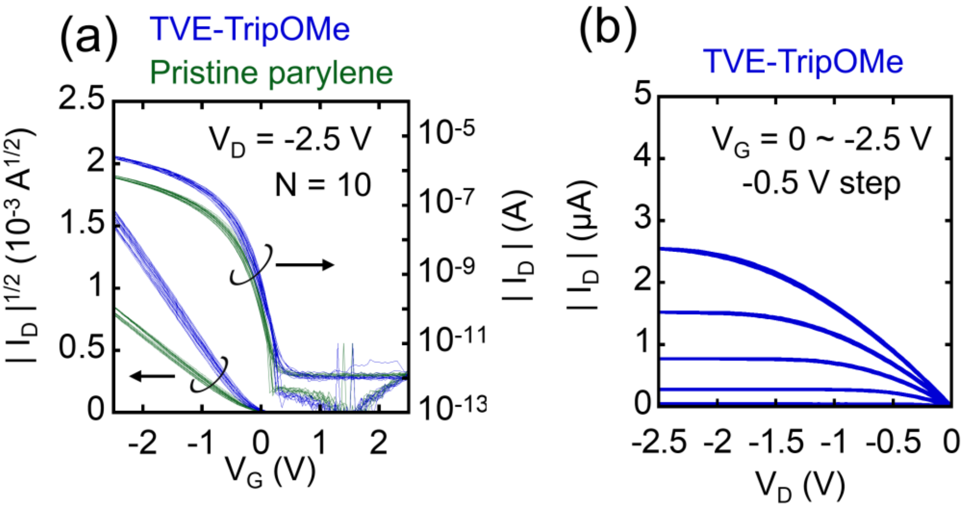

Figure S2. Electrical characteristics of OTFTs with TVE-TripOMe. a) Transfer characteristics in saturation regime. The green curves correspond to those OTFTs with pristine parylene, while the blue curves are those OTFTs on TVE-TripOMe. b) Output characteristics of OTFTs with TVE-TripOMe.

Mobility extraction and calculation of reliable factor (r)

In this study, we extracted mobility (μ) in saturation regime based on basic gradual channel approximation. In this approximation, the mobility can be expressed as:

$$\mu = \frac{2L}{WC_i}\left(\frac{\partial\sqrt{|I_D|}}{\partial|V_G|}\right)^2 \quad , \qquad (1)$$

where L and W are the channel length and channel width, C_i is the gate channel capacitance per unit area, I_D is the source-drain current, and V_G is the voltage in the gate terminal. As described in the main text, C_i was estimated from the capacitance of the metal/dielectric/metal structures. The channel length and width are described in *Fabrication and measurement of OTFTs.* To extract the mobility and threshold voltage, we applied fitting curves to the $\sqrt{I_D}$-V_G transfer characteristics to determine the slope. With regard to the fitting range, some data points around small ranges were selected at the intermediated V_G.

Based on the above-mentioned method, we estimated the reliability factor (r) for the extracted mobility of OTFTs with TVE-TripOMe and BC-TripOMe. In modified definition, r can be expressed as:[1]

$$r = \frac{\left(\frac{\sqrt{|I_D|_{V_G=V_GMAX}}-\sqrt{|I_D|_{V_G=V_{th}}}}{|V_{GMAX}-V_{th}|}\right)^2}{\left(\frac{\partial\sqrt{|I_D|}}{\partial|V_G|}\right)^2} \quad . \qquad (2)$$

The denominator expresses the square of the slope of the fitting curve to extract the mobility. The numerator expresses the square of the slope of the transfer curve of the electrically equivalent TFT in ideal conditions. V_{GMAX} is the maximum gate voltage to get the transfer characteristics. In this report, V_{GMAX} is -2.5 V. $|I_D|_{VG\ =VGMAX}$ and $|I_D|_{VG\ =Vth}$ are the source-drain current at the V_{GMAX} and at the V_{th}, respectively. As examples of the fitting curves for

extracting the mobility, the slope of the transfer curve of the electrically equivalent TFT under ideal conditions, and the corresponding r, are shown in Supplementary Figure S3. The estimated r for those with TVE-TripOMe and BC-Trip.OMe are 90 and 90%, respectively. These values suggest the high reliability of the mobility extraction in the saturation regime. In the main manuscript, we showed the averaged r for 10 OTFTs, as used in Figure 2d.

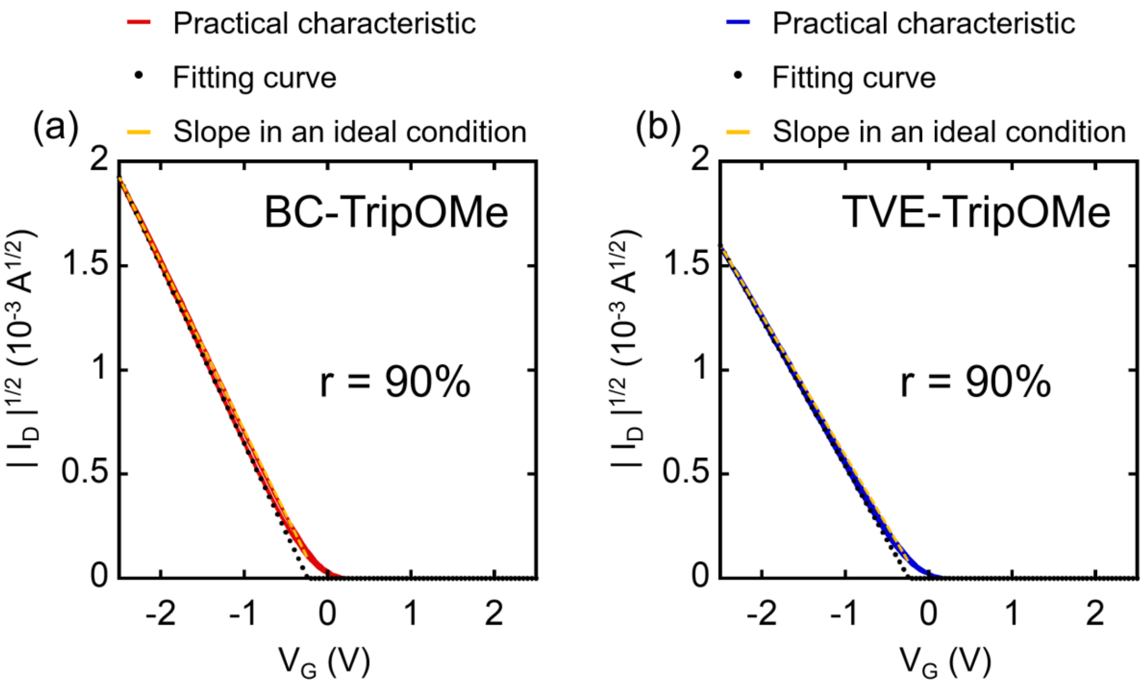

Figure S3. Comparison of fitting slope used to extract mobility with that for ideal conditions, used to estimate r. a) OTFT with BC-TripOMe. b) OTFT with TVE-TripOMe.

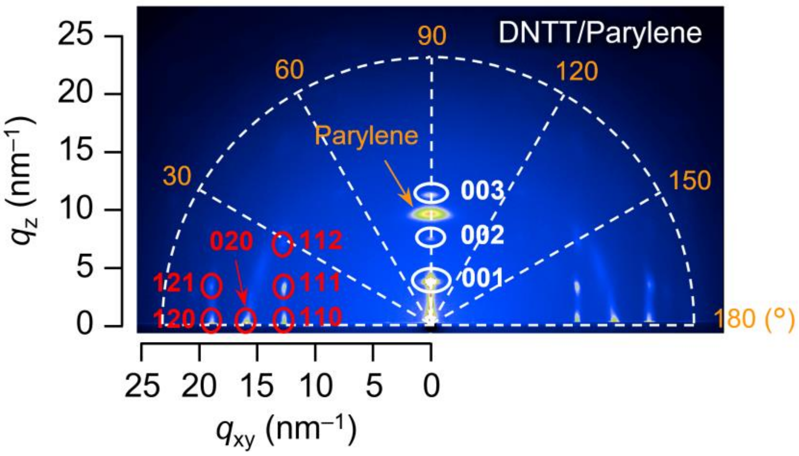

Figure S4. 2D grazing incidence X-ray diffraction (GI-XRD) pattern for DNTT on pristine parylene.

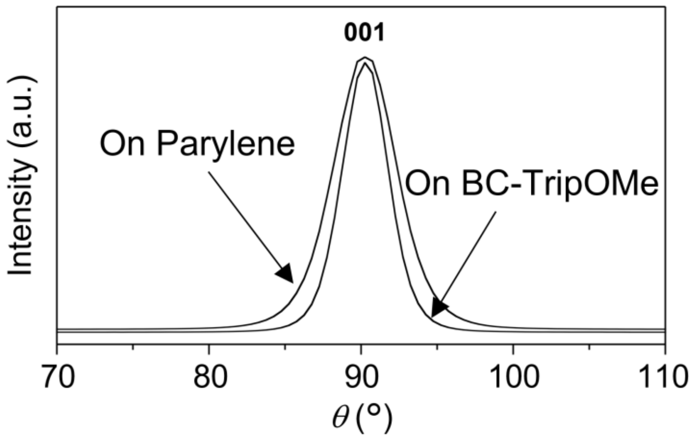

Figure S5. Comparison of azimuthal-angle dependency of the diffraction peak intensity of the (001) plane of DNTT on pristine parylene and on BC-TripOMe.

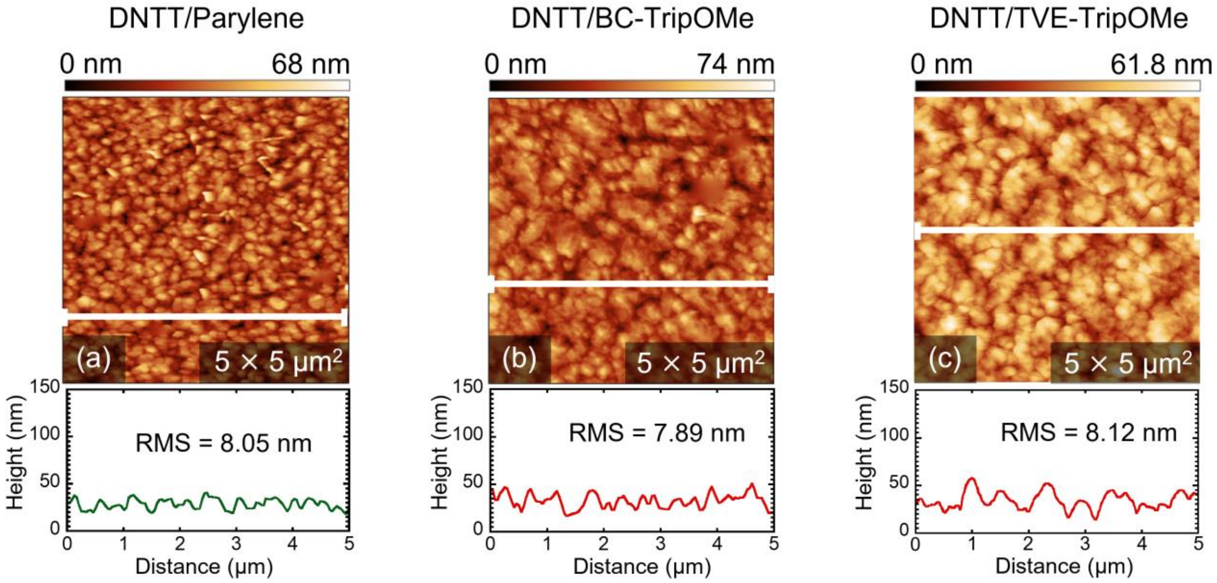

Figure S6. Morphologies of DNTT and the corresponding line profiles on a) pristine parylene, b) BC-TripOMe and c) TVE-TripOMe, respectively.

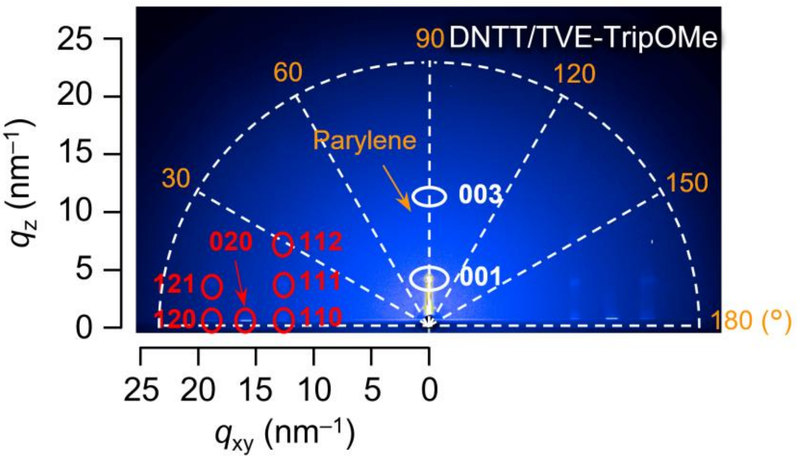

Figure S7. GI-XRD analysis of DNT films on TVE-TripOMe.

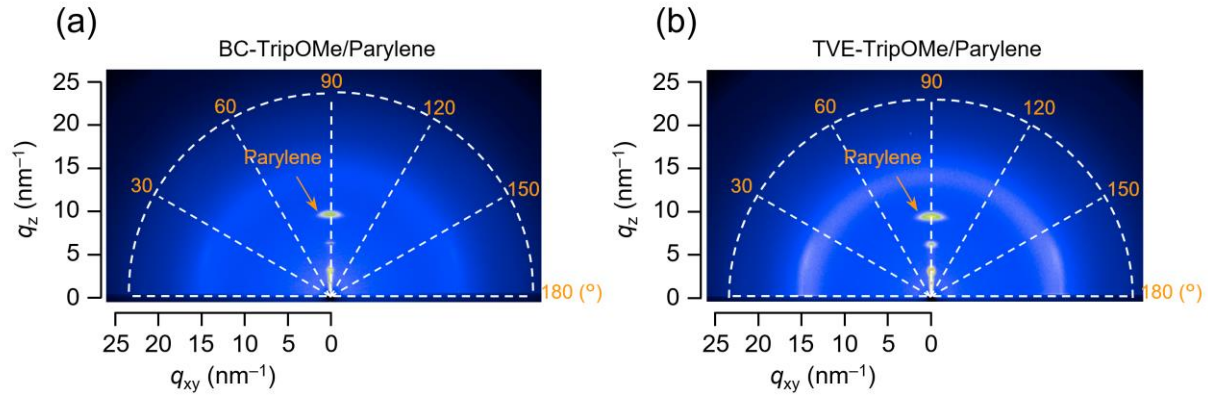

Figure S8. GI-XRD analysis of TripOMe films on parylene. a) BC-TripOMe. b) TVE-TripOMe.

Structural effect of Trip derivatives

We fabricated and measured OTFTs with a TripH modification layer formed by the TVE and BC processes (TVE-TripH and BC-TripH). The results are shown in Supplementary Figure S9. Note that the process batch is the same as that for OTFTs with a TripOMe modification layer except for the formation of the Trip layer. Supplementary Figure S9a shows mobility histograms of the OTFTs with TripH. The average mobility values of OTFTs with TVE- and BC-TripH are 0.18 ± 0.01 and 0.32 ± 0.03 cm^2/Vs, respectively. The OTFTs with BC-TripH exhibited a 40 % higher mobility than those with TVE-TripH. This trend is the same as for TripOMe. We conducted GI-XRD on a 30-nm DNTT film deposited on parylene and treated with TripH. Supplementary Figure S10a shows the results of 2D GI-XRD for a DNTT/BC-TripH/parylene film. Supplementary Figure S10b shows the 1D put-of-plane and in-plane diffraction peak profiles of DNTT on BC- and TVE-TripH. The peaks for DNTT on BC-TripH are shaper that those for TVE-TripH. This trend is consistent with the results of TripOMe in Figure 3b (see main text). This indicates that BC is the preferable process, irrespective of the types of Trip derivatives.

However, we noted that both the TVE- and BC-TripH decreased the OTFTs performance comparing with OTFTs with pristine parylene. First, we examined the reason of deteriorated mobility. We compared the FWHM of the azimuthal angle dependency of the diffraction spots indexed to the DNTT (001) plane with that of DNTT/BC-TripOMe/parylene film, as shown in Supplementary Figure S10c. The FWHM on BC-TripH was found to be larger than that on BC-TripOMe. As discussed in the main text, this result indicates that crystallites of DNTT on BC-TripH lower crystallinity relative to BC-TripOMe. In other words, DNTT on BC-TripH exhibits more disorders than that on BC-TripOMe. In addition, an AFM image revealed that DNTT on BC-TripH was composed of smaller crystalline structures than either pristine parylene or BC-TripOMe as shown in Supplementary Figures S6 and S11. Thus, the

smaller and more-disordered DNTT grain on TripH could be the main reason why OTFTs with TripH exhibit a lower mobility.

Second, to investigate the origin of the smaller and more-disordered DNTT grains on TripH, we observed the morphology of TripH films by using AFM. Supplementary Figure S12a,b shows the morphology of TVE- and BC-TripH, respectively. The RMS were 5.09 and 1.94 nm, respectively. In the TVE process, many small terraces with a large step of around 10 nm were formed. On the other hand, the BC process dramatically improved the surface morphology of TripH. In addition, it should be emphasized that the surface of TripH has a lower surface energy than that of TripOMe (see Supplementary Table S2). However, the use of TripH led to smaller and more-disordered DNTT grains. As discussed in the main manuscript, the phase states (that is, the packing) of SAMs also influence the growth of organic semiconductors. These effects of TripH imply that TripH could present a greater degree of undesirable packing than TripOMe for the growth of organic semiconductors. Surprisingly, the difference between TripH and TripOMe is only a methoxy group. This result is important not only for synthesizing new Trip derivatives but also for establishing a deeper understanding of the effect of the phase states in the self-assembled layers of organic devices.

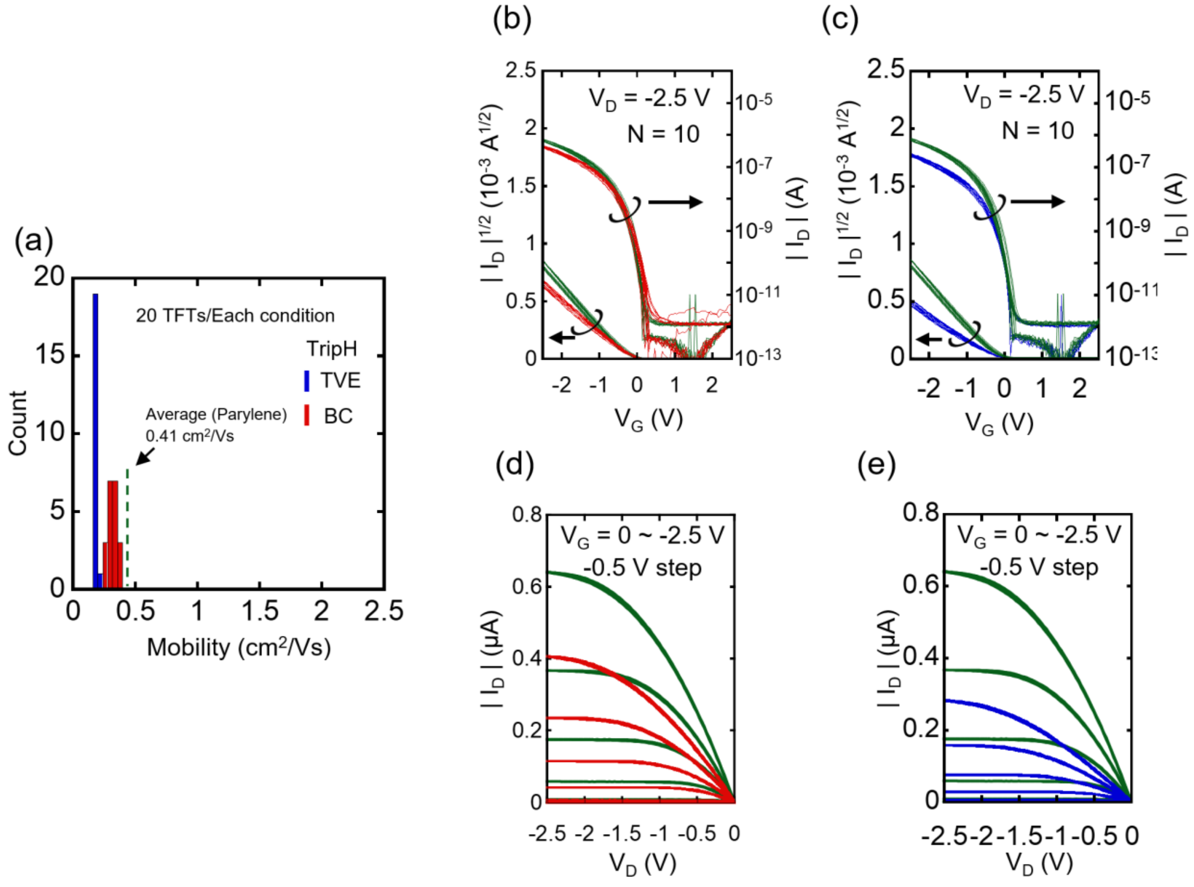

Figure S9. Electrical characteristics of OTFTs with TripH. a) Histograms of field-effect mobility of OTFTs. The blue histograms show those OTFTs with TVE-TripH films. The red histogram shows those OTFTs with BC-TripH films. b) Transfer characteristics in saturation regime. The green curves correspond to OTFTs with pristine parylene while the red curves correspond to the OTFTs with BC-TripH. c) Transfer characteristics in saturation regime. The green curves correspond to those OTFTs with pristine parylene while the blue curves correspond to the OTFTs with TVE-TripH. d) Output characteristics. The green curve corresponds to those OTFTs with pristine parylene while the red curve corresponds to the OTFTs with BC-TripH formed. e) Output characteristics. The green curve corresponds to those OTFTs with pristine parylene while the blue curve corresponds to OTFTs with TVE-TripH.

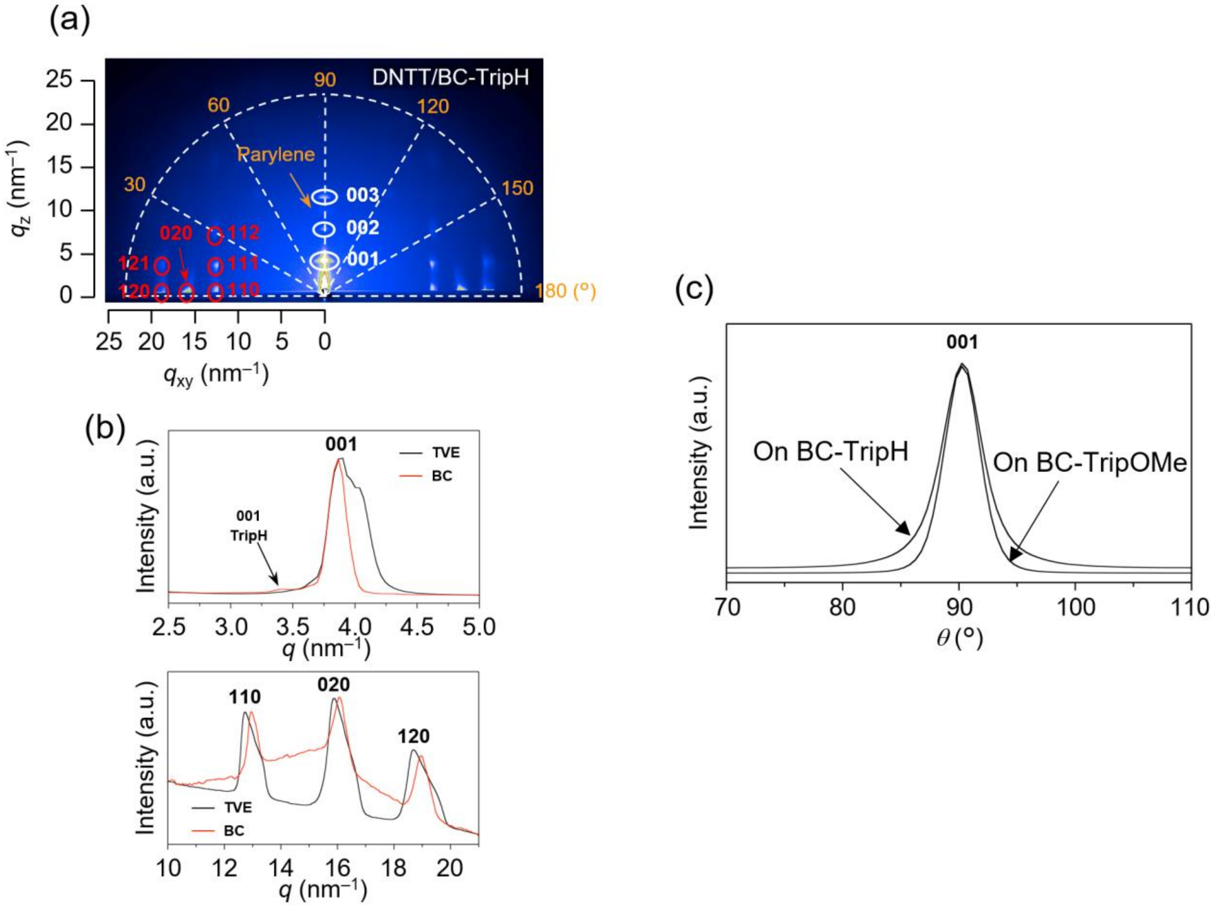

Figure S10. GI-XRD analysis of 30-nm DNTT layer on TripH. a) 2D GI-XRD pattern of DNTT on BC-TripH. b) 1D GI-XRD profiles of DNTT films on TVE-TripH (black line) and BC-TripH (red line). c) Comparison of azimuthal-angle dependency of the diffraction peak intensity from the (001) plane of DNTT on BC-TripOMe and BC-TripH.

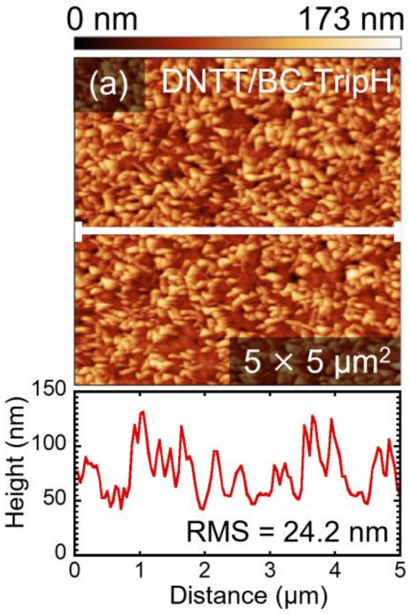

Figure S11. Morphology of DNTT on BC-TripH and corresponding line profiles.

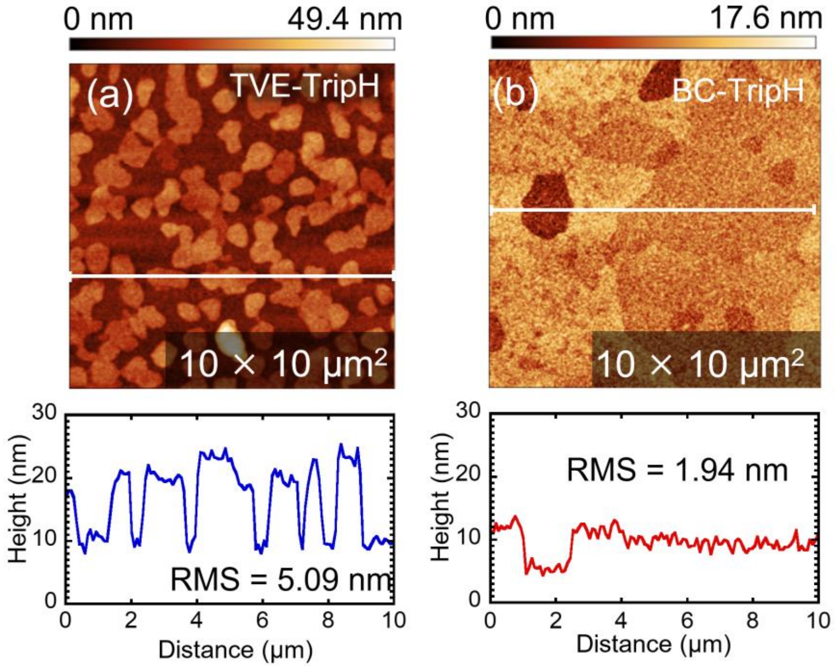

Figure S12. Morphology of TripH and corresponding line profiles. a) TVE-TripH. b) BC-TripH.

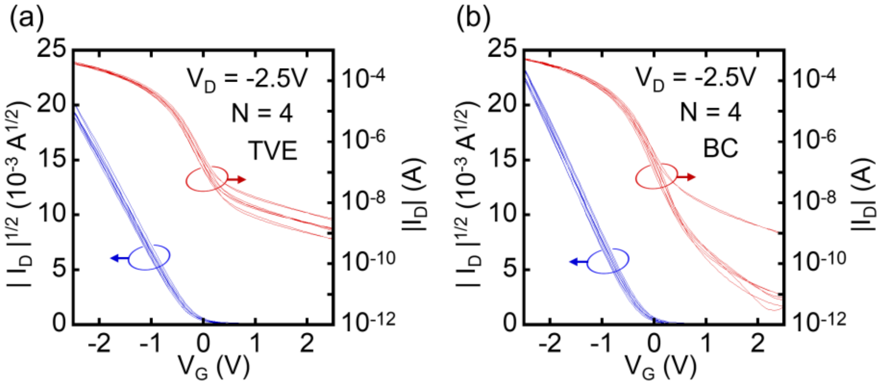

	(a) TVE-TripOMe	(b) BC-TripOMe
Mobility (cm^2/Vs)	1.64 ± 0.06	2.13 ± 0.05
V_{th} (V)	-0.315 ± 0.016	-0.323 ± 0.030

Figure S13. Comparison of characteristics of OTFTs with a) TVE-TripOMe and b) BC-TripOMe, respectively. The channel length and width of the OTFTs are 10 μm and 16 mm, respectively. The transistor size is larger than that used in Figure 2.

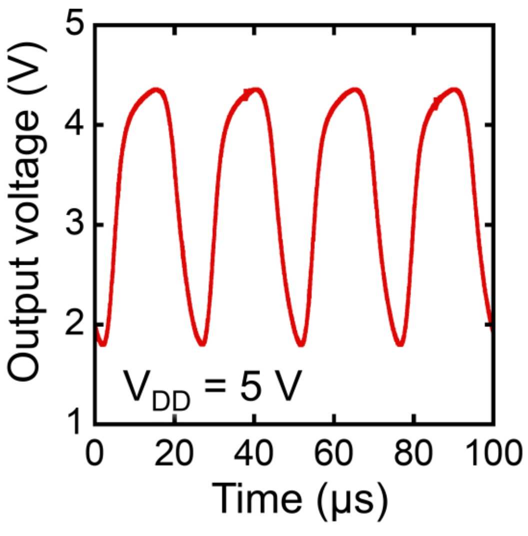

Figure S14. Typical output signal of organic ring oscillator with BC-TripOMe.

	a [Å]	*b* [Å]	*c* [Å]	*β* [°]	*V* [Å^3]
Pristine parylene	6.21	7.82	16.20	87.29	785.26
TVE-TripH	6.34	7.92	16.42	87.00	823.49
BC-TripH	6.21	7.82	16.36	86.71	793.38
TVE-TripOMe	6.34	7.93	16.35	87.45	821.15
BC-TripOMe	6.15	7.80	16.13	87.81	774.33

Table S1
Lattice constant of DNTT on parylene SR with and without triptycene derivatives

	γ_S^p [mJ/m^2]	γ_S^d [mJ/m^2]	γ_S [mJ/m^2]
Pristine parylene	5.8 ± 0.4	20.3 ± 0.3	26.0 ± 0.3
TVE-TripH	1.1 ± 0.3	20.3 ± 0.7	20.1 ± 0.8
TVE-TripOMe	0.91 ± 0.21	21.8 ± 0.4	22.7 ± 0.2
BC-TripH	1.3 ± 0.6	22.1 ± 0.6	20.7 ± 1.6
BC-TripOMe	0.88 ± 0.04	21.5 ± 0.4	22.4 ± 0.4

Table S2
Surface energy of parylene with/without triptycene derivatives

References

[1] Xu, Y. *et al.* Essential effects on the mobility extraction reliability for organic transistors. *Adv. Funct. Mater.* 1803907 (2018).
